# The quality of life in Chinese juvenile idiopathic arthritis patients: psychometric properties of the pediatric quality of life inventor generic core scales and rheumatology module

**DOI:** 10.1186/s12955-021-01683-2

**Published:** 2021-01-30

**Authors:** Hua-hong Wu, Feng-qi Wu, Yang Li, Jian-ming Lai, Gai-xiu Su, Shu-hua Cui, Zheng Chen, Hui Li

**Affiliations:** 1 Department of Growth and Development, Capital Institute of Pediatrics, YaBao Road, ChaoYang District, Beijing, 100020 China; 2 Department of Rheumatism and Immunology, Children’s Hospital Affiliated to Capital Institute of Pediatrics, Beijing, China; 3 Hebei Yanda Hospital, Yanjiao, Hebei China

## Abstract

**Background:**

Juvenile idiopathic arthritis (JIA) may seriously affects patients’ quality of life (QoL), but it was rarely focused and studied in China, so we explore JIA children’s QoL using Chinese version of the PedsQL4.0 Generic Core and PedsQL3.0 Rheumatology Module scale, and analyzed the psychometric properties of these two Scales among Chinese JIA children.

**Methods:**

We recruited 180 JIA patients from Children's Hospital Affiliated to Capital Institute of Pediatrics and Hebei Yanda Hospital from July 2018 to August 2019. The questionnaires include information related on JIA, PedsQL4.0 generic core and PedsQL3.0 Rheumatology Module scales. According to the disease type, onset age of and course of JIA, we divided them into different groups, then compared the QoL status among different groups. Moreover, we analyzed the reliability and validity of these two scales in these 180 JIA children.

**Results:**

The mean score of PedsQL4.0 generic core scale on these 180 patients was 82.85 ± 14.82, for these in active period was 72.05 ± 15.29, in remission period was 89.77 ± 9.23; the QoL score of systemic, polyarticular and oligoarticular JIA patients were 77.05 ± 19.11, 84.33 ± 12.46 and 87.12 ± 10.23. The mean score of PedsQL3.0 Rheumatology Module scale on 180 patients was 91.22 ± 9.45, for these in active period was 84.70 ± 11.37, in remission period was 95.43 ± 4.48; the QoL score of systemic, polyarticular and oligoarticular JIA patients were 89.41 ± 11.54, 89.38 ± 10.08 and 93.71 ± 6.92. In the PedsQL 4.0 Generic Core scale, the α coefficients of total scale and almost every dimension are all greater than 0.8 except for the school activity dimension of 0.589; the correlation coefficients of 22 items’ scores (total 23 items) with the scores of dimensions they belong to are greater than 0.5 (maximum value is 0.864), and the other one is 0.406. In PedsQL3.0 Rheumatology Module scale, except for the treatment and worry dimensions of 0.652 and 0.635, the α coefficients of other dimensions and the total scale are all greater than 0.7; the correlation coefficients of all items’ score were greater than 0.5 (the maximum is 0.933, the minimum is 0.515).

**Conclusions:**

The QoL of Chinese JIA children is worse than their healthy peers, these in active period and diagnosed as systemic type were undergoing worst quality of life. The reliability and validity of PedsQL 4.0 Generic Core and PedsQL3.0 Rheumatology Module scale in Chinese JIA children are satisfactory, and can be used in clinical and scientific researches.

## Background

Juvenile idiopathic arthritis (JIA) is a systemic chronic inflammatory disease occurred in children below 16 years, featured on chronic joint synovitis and extra articular organs’ damages. It is the most common type of rheumatic diseases in children with prevalence varies from 4.01 to 70 ‰ [1]. During the active period of JIA, children may suffer from high fever, general malaise, joint swelling and pain, and limited daily activities every day [2]. Although there are already great progress on the treatment of JIA in recent years, it is still a chronic persistent disease and have high recurrence rate, even in remission period it still needs drug maintenance therapy, and some severe cases cannot able to be cured. The pain, disability, psychological pressure and treatment discomfort caused by long-term joint and muscle damage all have impact on the physiological, psychological and self-awareness of patients [3, 4]. Some children are even out of school, depressed, and with declined social adaptability, all of which seriously affects their quality of life (QoL) [5]. So, for JIA patients, traditional evaluation indexes such as mortality, remission rate and effective rate cannot fully evaluate the effect of intervention measures, and cannot meet the new requirements of people for health. Therefore, the evaluation of QoL has become one of the indispensable evaluation contents in the study of chronic diseases such as rheumatism.

The QoL scale for JIA patients and related studies were very mature in many countries [3–5], but there are few researches on that in China. The Pediatric Quality of Life Inventory (Peds QL) developed by Varni contains a series of scales and is the most widely used scale for children's QoL until now. Among which, the PedsQL4.0 Generic Core Scales (PedsQL 4.0-GC**)** can be applied to all children, and the PedsQL3.0 Rheumatology Module (PedsQL3.0-RM) was specifically tailored for pediatric rheumatology [6]. These two scales have both been translated into Chinese version [7, 8], but their application on JIA was hardly seen in China. So, in this study, we applied the Chinese version of PedsQL 4.0-GC and PedsQL3.0-RM, to evaluated the status and influencing factors of the QoL in Chinese JIA children, and explored the psychometric properties of these two scales in these children, and provide basis for further research and improvement on the QoL of JIA children.

## Material and methods

### Subjects

All subjects were recruited from rheumatic immune clinic of Children's Hospital Affiliated to Capital Institute of Pediatrics and Pediatric clinic of Hebei Yanda Hospital from July 2018 to August 2019. Inclusion criteria: (1) JIA patients diagnosed in line with the standard of International Association of Rheumatology [9]. (2) The course of JIA is longer than three months, patients and/or their parents have a certain understanding of JIA. (3) Patients aged 8–18 years and have normal understanding and expression ability. Exclusion criteria: (1) JIA patients complied with other serious diseases affected the QoL of children, such as serious infection. (2) Failed to understand the scale items or cannot answer the items correctly.

### Questionnaire and scales

Questionnaire consisted of general information such as gender, age, onset age of JIA, diagnosis age, disease type, treatment history, disease activity, etc.

Chinese Version of the PedsQL 4.0-GC and PedsQL3.0-RM was applied to evaluate children’s QoL. PedsQL 4.0-GC scale was composed of 23 items which divided into 4 dimensions, physical health (8 items), emotional functioning (5 items), social functioning (5 items) and school functioning (5 items). PedsQL3.0-RM composed of 22 items which divided into 5 dimensions, pain and hurt (4 items), daily activities (5 items), treatment (7 items), worry (3 items), and communication (3 items).

### Data collecting and analysis

The questionnaire and PedsQL scales were all interview-administered one by one. In this paper, we only studied the child self-reports of PedsQL 4.0-GC and PedsQL3.0-RM which were filled solely on children’s answers.

In order to analyze the influencing factors of JIA patients’ QoL, we divided all subjects into different groups: according the onset age of JIA to four groups (< 5 years, 5 ~ 8 years, 8 ~ 12 years and > 12 years); according the course to three groups (< 3 years, 3 ~ 5 years and > 5 years); according to the disease type to three groups (Systemic, Polyarticular and Oligoarticular); according to activity of JIA to two groups (active and remission). Criteria for determining the disease in remission period were no active joint inflammation; no fever, rash, serositis and other systemic symptoms; no active ophthalmic diseases; ESR and CRP are in the normal range; the overall assessment of the disease by pediatric rheumatologist concluded that it is not in active period [10].

### Statistics

The data were analyzed by SPSS 16.0 software. The total score and each dimension’s score of PedsQL 4.0-GC and PedsQL3.0-RM were described by mean ± SD. The differences of scores among different groups were analyzed using con-variable test or independent sample t-tests. *P* < 0.05 was considered statistically significant. The reliability and validity of PedsQL 4.0-GC and PedsQL3.0-RM scale were calculated by Cronbach’sαcoefficients and Pearson correlation coefficients. In general, a Cronbach’s α coefficient ranging from 0.70 to 0.84 is regarded as satisfactory inter consistence. Pearson’s Correlation coefficient effect sizes are designated as small (0.10–0.29), medium (0.30–0.49), and large (0.50 or more) in magnitude.

### Ethical approval

This study was approved by Ethics Committee of Capital institute of pediatrics.

## Results

A total of 180 JIA children (102 boys and 78 girls) aged 8 ~ 18 years participated in this survey. The general information such as age, onset age of disease, course and drug application was showed in Table 1.

**Table 1:** The basic information of JIA patients

	N	Min	Max	Mean	SD
Age (y)	179	8.00	18.22	10.47	2.66
Onset age of JIA (y)	160	0.50	14.90	6.81	3.10
Diagnosis age (y)	170	0.50	15.10	7.43	3.04
Course of JIA (y)	160	0.00	14.3	3.57	2.55

### The Qol of JIA patients and differences between active and remission period

We calculate the total score and each dimension of PedsQL 4.0-GC and PedsQL3.0-RM for these 180 children, and QoL scores of patients in active and remission period respectively. The results are shown in Table 2. The QoL score of patients in remission period was higher than those in active period, the mean score of PedsQL 4.0-GC score was 17.72 points higher, and that of PedsQL3.0-RM scale was 10.73 points higher. The differences on total score and every dimension between active and remission period are all significant (*P* < 0.05).

**Table 2:** The Qol of 180 patients and differences between active and remission period

	Total (n = 180)	Active (n = 72)	Remission (n = 108)	*F*	*P*
*PedsQL 4.0-GC*					
Total score (23 items)	82.85 ± 14.82	72.05 ± 15.29	89.77 ± 9.23	93.426	0.000
physical functioning (8 items)	82.58 ± 19.63	67.16 ± 20.78	92.63 ± 9.77	116.443	0.000
emotional functioning (5 items)	89.06 ± 15.54	82.10 ± 18.38	93.48 ± 11.50	25.230	0.000
social functioning (5 items)	87.93 ± 17.16	78.24 ± 20.70	94.41 ± 10.06	46.550	0.000
school functioning (5 items)	74.28 ± 16.14	65.88 ± 16.99	79.32 ± 12.99	34.200	0.000
*PedsQL3.0-RM*					
Total score (22 items)	91.22 ± 9.45	84.70 ± 11.37	95.43 ± 4.48	74.983	0.000
pain and hurt (4 items)	86.07 ± 18.42	72.61 ± 21.02	95.07 ± 8.74	94.100	0.000
daily activities (5 items)	98.45 ± 7.91	96.34 ± 12.42	99.76 ± 1.28	7.767	0.006
Treatment (7 items)	90.72 ± 10.78	83.96 ± 12.71	95.01 ± 6.57	54.144	0.000
Worry (3 items)	91.96 ± 12.98	87.95 ± 15.38	94.55 ± 10.50	10.812	0.001
Communication (3 items)	87.35 ± 17.58	81.69 ± 21.09	90.76 ± 14.04	11.135	0.001

### The QoL of JIA patients in different types

Table 3 showed the QoL scores of JIA patients in three disease types. We can see that the QoL score of Oligoarticular, Polyarticular and Systemic patients are gradually reduced. In PedsQL 4.0-GC, the differences between these three types were all significant except the emotional dimension. There are the same trend in PedsQL3.0-RM scale: with the lowest score of children in systemic type and the highest score of children in Oligoarticular type, but there was no significant difference between different types in pain, daily activities and worry dimensions.

**Table 3:** The Qol scores of JIA patients in different types

	Systemic (n = 60)	Polyarticular (n = 70)	Oligoarticular (n = 38)	*F*	*P*
*PedsQL 4.0-GC*					
Total score (24 items)	77.05 ± 19.11	84.33 ± 12.46	87.12 ± 10.23	5.435	0.001
Physical functioning (8 items)	76.83 ± 23.67	84.38 ± 20.40	86.88 ± 15.11	2.826	0.040
Emotional functioning (5 items)	87.28 ± 18.76	87.70 ± 14.12	92.65 ± 12.02	1.686	0.172
Social functioning (5 items)	81.34 ± 21.33	89.17 ± 15.88	92.97 ± 11.98	5.764	0.001
School functioning (5 items)	67.64 ± 18.83	77.03 ± 14.02	77.28 ± 13.99	4.728	0.003
*PedsQL3.0-RM*					
Total score (22 items)	89.41 ± 11.54	89.38 ± 10.08	93.71 ± 6.92	3.007	0.032
Pain and hurt (4 items)	82.73 ± 20.55	82.73 ± 19.89	90.07 ± 13.92	2.421	0.068
Daily activities (5 items)	97.50 ± 10.85	97.50 ± 9.98	99.41 ± 3.29	0.716	0.544
Treatment (7 items)	88.69 ± 12.60	88.72 ± 12.85	93.65 ± 7.16	3.679	0.014
Worry (3 items)	91.82 ± 13.00	89.86 ± 13.77	93.75 ± 12.06	0.737	0.531
Communication (3 items)	85.03 ± 21.80	85.96 ± 15.87	91.41 ± 12.86	3.177	0.026

### The impact of onset age and course of JIA on patients’ QoL

**Fig. 1:**
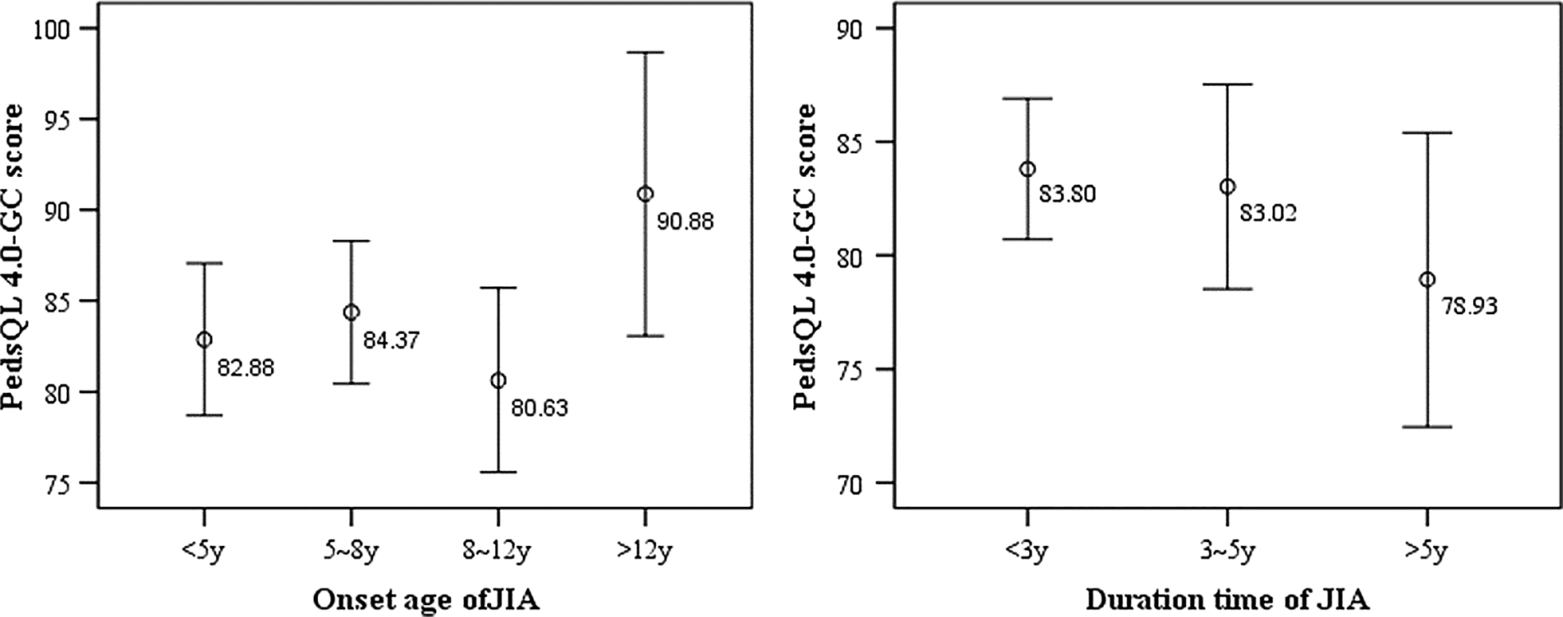
The PedsQL 4.0-GC scores of children in different groups. *Note*: the numbers were the mean QoL score of each group

**Fig. 2:**
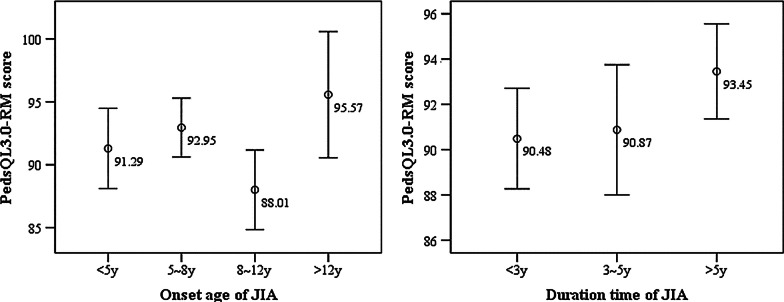
The PedsQL3.0-RM scores of children in different groups. *Note*: the numbers were the mean QoL score of each group

According to the classification of the onset age and course of JIA, in this survey, 45 children with onset age younger than 5 years, 56 children’s onset age are between 5 and 8 years, 53 children between 8 and 12 years, 6 children older than 12 years; 101 children’s course shorter than 3 years, 36 children’s courses between 3 and 5 years and 32 children’s course longer than 5 years. We calculated the QoL scores of different kinds of JIA patients. As shown in Fig. 1, the PedsQL 4.0-GC scores of children with onset age older than 12 years were higher than other groups, but the difference was not statistically significant (*F* = 1.091, *P* = 0.355). The scores of children whose course longer than 5 years were slightly lower than other groups, and the difference also no statistical significance (*F* = 1.152, *P* = 0.319). In Fig. 2, the PedsQL3.0-RM score of children with onset age older than 12 years was also higher than other groups, the difference was statistically significant (*F* = 2.709, *P* = 0.047), and there was no significant difference between groups with different course of JIA (*F* = 0.956, *P* = 0.387).

### Reliability and validity of the scales

The Cronbach's αcoefficient is used to evaluate the reliability of the scale. In the PedsQL 4.0-GC scale, theαcoefficients of total scale and almost every dimension are all greater than 0.8 except for the school activity dimension of 0.589; In PedsQL3.0-RM scale, except for the treatment and worry dimensions of 0.652 and 0.635, theαcoefficients of other dimensions and the total scale are all greater than 0.7, so the reliability of the scale is satisfactory.

The validity of the scale is expressed by the correlation between each item and the dimension it belonged. In PedsQL 4.0-GC scale, the correlation coefficients of 22 items are greater than 0.5 (maximum value is 0.864), and the other one is 0.406. In PedsQL3.0-RM scale, the correlation coefficients of all items is greater than 0.5 (the maximum is 0.933, the minimum is 0.515), which are all highly correlated.

## Discussion

The QoL of JIA children is worse than healthy children, and those in active period is significantly worse than those in remission period especially in dimensions of physiological function, school function and pain. In this study, the average score of PedsQL 4.0-GC scale is 82.85, the score of school function dimension is 74.28, which are lower than the scores of healthy children (87.36 and 81.29) in previous survey [11]. This is due to the impact of JIA on children's physical and psychosocial functions: the pain and different disability caused by JIA greatly limits children's daily activities, leading to their social activities decreased [12]; long-term therapy of immunosuppressant and glucocorticoid resulted in growth retardation, changes of children’s face and body shape, all of these induced a series of negative emotions such as inferiority, anxiety and depression [13]. Also, it may lead to memory decline and attention loss, seriously affect patients' academic performance and even lead to children dropping out of school. A global multicenter cohort study also found that the QoL of JIA children was significantly reduced especially the physical dimension, disability and pain were the most important factors affecting the physical dimension and psychosocial dimension respectively [14], and serious activity of JIA was the risk factor for poor QoL of children [15]. Feng's research also confirmed that the most important factors affecting the QoL of JIA children are physical pain and high-intensity exercise restriction, and the QoL damage is more serious in the active period of disease, which is consistent with clinical observation and previous studies [16–18].

The type of disease also can affect the QoL of JIA children. The QoL of patients in Oligoarticular type is the best, followed by patients in Polyarticular type, and those in systemic type is the worst. Table 3 showed that, the scores of PedsQL 4.0-GC and PedsQL3.0-RM scale in systemic JIA were 77.05 and 89.41, which were significantly lower than that of Oligoarticular JIA (87.12 and 93.71), and there were all significant differences between three types in five dimensions of physiological function, social interaction, school, treatment and communication. Bouchra amine also confirmed that compared with polyarticular and systemic JIA, the Oligoarticular JIA patients has better motor function, less psychosocial dysfunction and clinical symptoms, so their QoL were significantly better than other types [19]. The possible reasons are patients in polyarticular and systemic type suffered from more joints and systemic symptoms, which lead to limb pain, limited activity and reduced social participation. In addition, pain, rigidity and fatigue are the main reasons affect children's enrollment rate and social activity participation [12], and all these factors lead to a significant reduction in the QoL of children in systemic JIA.

For children with older onset age of JIA, their QoL were better than those with younger onset age. This is confirmed by the fact that the QoL score of children with onset age older than 12 years are significantly higher than other groups (Figs. 1, 2), suggesting that the younger the onset age is, the worse the prognosis of JIA and their QoL. There are inconsistent conclusions about the effect of course on the QoL of JIA children. Some studies considered short course is a risk factor for poor QoL [20], while others found the longer course lead to the worse QoL [19]. Our study does not find the effect of course on QoL of JIA children. These different conclusions may affected by other confounding factor such as disease activity, disability degree and disease type. Another important factor is the large-scale use of biological agents in recent years, which greatly reduces the risk of children's disability and limited activity due to long course of disease, ensures children's participation in social activities, all above comprehensively improve the QoL of JIA patients. However, for early-onset systemic JIA, the disease recurrence rate is relatively high, so early diagnosis and treatment is still one of the most important measure to improve their long-term QoL [21].

The reliability and validity of PedsQL 4.0-GC and PedsQL3.0-RM scale for Chinese JIA patients are all satisfactory, so can be used in clinical and scientific evaluation. The Cronbach's αcoefficient of these two scales is both greater than 0.7, which suggest greater inter consistencies. In PedsQL 4.0-GC scale, the correlation coefficients of 18 items and their dimensions is greater than 0.7, the other 4 items is between 0.5 and 0.7, only one item "I lost three and dropped four" is 0.406. In PedsQL3.0-RM scale, the correlation coefficient of all items and their dimensions is greater than 0.5, which suggest a good validity. At the same time, more and more people believe that it is necessary to evaluate the QoL of JIA children. Besides pain and disability, many factors such as age, cognitive level, understanding of JIA, parents' attitudes, family’s financial burden, school and social’s support may all affect the QoL of JIA children. For example, the long course of disease, higher recurrences and the biological agents therapy have brought great economic burden to families and society (A study of 162 JIA children in Europe shows that the medical cost per person per year ranges from 18,913 to 36,396 Euro [22]), and children may attacked by feelings of inferiority and guilt, even close or abandon themselves and lost their labor force, which will lead to greater socio-economic loss. Therefore, we need a standardized evaluation tool to accurately evaluate the QoL of JIA children. According to the results, we can give psychological intervention, exercise, social support and other combined interventions, and provide a comprehensive, multidisciplinary and practical individualized treatment plan for every JIA children.

## Conclusion

In conclusion, the QoL of JIA children in China is worse than their healthy peers, especially in physiological function and school function. The activity and type of JIA are important factors affecting those children’s QoL. The reliability and validity of PedsQL 4.0-GC and PedsQL3.0-RM scale in Chinese JIA children are satisfactory, and can be used in clinical and scientific research. This may help pediatric rheumatologist to pay more attention to the QoL of children when they treating their basic diseases, and provide basis for development of multi-disciplinary individual intervention programs in order to practical improve the QoL of JIA children in China.

## Abbreviations

JIA: Juvenile idiopathic arthritis
QoL: Quality of life
PedsQL 4.0-GC: Pediatric Quality of Life Inventory 4.0 Generic Core Scales
PedsQL3.0-RM: Pediatric Quality of Life Inventory 3.0 Rheumatology Module
